# Complete Genome Sequence of Rhodococcus erythropolis X5, a Psychrotrophic Hydrocarbon-Degrading Biosurfactant-Producing Bacterium

**DOI:** 10.1128/MRA.01234-19

**Published:** 2019-11-27

**Authors:** Yanina Delegan, Leonid Valentovich, Kirill Petrikov, Anna Vetrova, Artur Akhremchuk, Vladimir Akimov

**Affiliations:** a Institute of Biochemistry and Physiology of Microorganisms, Pushchino, Russian Federation; b Federal Research Center–Pushchino Scientific Center for Biological Research of the Russian Academy of Sciences, Pushchino, Russian Federation; c Institute of Microbiology of the National Academy of Sciences of Belarus, Minsk, Belarus; University of Arizona

## Abstract

Rhodococcus erythropolis X5 is a psychrotrophic (cold-adapted) hydrocarbon-degrading bacterium, as it showed effective *n*-alkane destruction at low positive temperatures. Here, the genome of strain X5 was completely sequenced; it consists of a 6,472,161-bp circular chromosome (62.25% GC content) and a 526,979-bp linear plasmid, pRhX5-526k (62.37% GC content).

## ANNOUNCEMENT

Rhodococci are typical representatives of soil microflora (1). These actinobacteria are well known as metabolically versatile microorganisms with potential applications in bioremediation (2). The strain Rhodococcus erythropolis X5 (VKM Ac-2532D) was isolated from oil-polluted soil (3). R. erythropolis X5 is a psychrotrophic (cold-adapted) hydrocarbon-degrading bacterium, as it showed effective *n*-alkane destruction at low positive temperatures (3). The strain is a biosurfactant producer; during growth on hydrocarbon substrates, it forms extracellular succinoyl trehalose lipids (4). For long-term storage, the strain was kept in glycerol (40%) stocks at −70°C. For short-term maintenance and biomass preparation, the strain was cultured on a lysogeny broth (LB) agar plate at 27°C.

Genomic DNA was isolated from a fresh culture biomass (a colony) of Rhodococcus erythropolis X5 grown on LB agar using a DNeasy blood and tissue kit (catalog number 69506; Qiagen). Sequencing was performed using a MinIon sequencer (Oxford Nanopore Technologies [ONT]) at the Center of Analytical and Genetic Engineering Research (Minsk, Belarus). A library was prepared with the ONT ligation sequencing kit (catalog number SQK-LSK109). Guppy v3.2.4 software was used for base calling, which yielded a total of 301 Mbp, distributed in 33,708 reads. Reads with a Q score of >10 were used for further analysis. Additionally, the same DNA sample was sequenced with an Illumina MiSeq platform using a MiSeq reagent kit v3 (2 × 300 bp). A paired-end library for sequencing was prepared with the MuSeek library preparation kit (catalog number K1361; Thermo Fisher). The Nanopore reads were assembled into 2 contigs using Canu assembler v1.8 (5). The Illumina reads were used to correct Nanopore errors using Bowtie 2 v2.3.5.1 (6) and Pilon v1.23 (7) software. Default parameters were used for all software.

The X5 genome consists of a 6,472,161-bp circular chromosome (62.25% GC content) and a 526,979-bp linear plasmid, pRhX5-526k (62.37% GC content). Chromosome circularization and plasmid linearity were specified by Canu. Also, there were no reads overlapping with plasmid ends. The strain was previously identified as R. erythropolis. For the average nucleotide identity (ANI) analysis, we used all available GenBank data on the R. erythropolis representatives and on Rhodococcus sp. strains. Average nucleotide identity parameter analysis revealed that the closest phylogenetic relatives of strain X5 are *Rhodococcus* sp. strain 008 (GenBank accession number CP012749), *Rhodococcus* sp. strain NJ-530 (CP034152), R. erythropolis CCM2595 (CP003761), and *Rhodococcus* sp. strain YL-1 (CP017299).

The X5 genome was annotated with the NCBI Prokaryotic Genome Annotation Pipeline (PGAP) v4.6 (8), which identified 6,305 coding sequences, 5 rRNA clusters (5S, 16S, and 23S), and 53 tRNAs. It was revealed that the genome of R. erythropolis X5 bears 5 copies of alkane hydroxylase-encoding gene *alkB*. These genes are essential for the degradation process of alkanes with a chain length of C_12_ to C_20_ (9, 10). In addition, 3 copies of a gene encoding cytochrome P450 hydroxylase were found on the plasmid. The genes encoding P450 hydroxylases are responsible for degradation of short alkanes (11, 12). Apart from that, the X5 plasmid bears a number of genes responsible for metal resistance. An antiSMASH search for secondary metabolite clusters found 17 functional clusters on the chromosome, including clusters for heterobactin, ectoine, and erythrochelin production. The genome sequence of R. erythropolis X5 provides essential data on alkane degradation and biosurfactant production by cold-adapted microorganisms.

### Data availability.

This genome project has been deposited in the NCBI database under GenBank accession numbers CP044283 and CP044284, BioSample number SAMN12818508, BioProject number PRJNA573614, and SRA accession number PRJNA573614.
